# Supplementary material for: Stable isotope analyses reveal unique trophic role of reef manta rays (Mobula alfredi) at a remote coral reef
Source: R Soc Open Sci. 2019 Sep 11;6(9):190599. doi: 10.1098/rsos.190599 (PMC6774984; doi:10.1098/rsos.190599)
Supplement: Supplementary Materials [file rsos190599supp1.docx]

*The following supplement accompanies the article*

**Stable isotope analyses reveal unique trophic role of reef manta rays (*Mobula alfredi*) at a remote coral reef**

Lauren R Peel^*^, Ryan Daly, Clare A Keating Daly, Guy MW Stevens, Shaun P Collin, Mark G Meekan

*Corresponding author: [lauren.peel@research.uwa.edu.au](mailto:lauren.peel@research.uwa.edu.au)

**Table S1** Summary of reef fishes sampled at D’Arros Island and St. Joseph Atoll, Seychelles, for stable isotope analysis. Estimated trophic level (TL; [www.fishbase.org](http://www.fishbase.org), 29/10/2018) and fork length given as mean ± standard error.

| **Trophic Guild** | **Species Name** | **n** | **Est. TL** | **Fork Length (cm)** | **Capture Method (# Samples)** |
| --- | --- | --- | --- | --- | --- |
| Herbivore | *Chlorurus sordidus* | 10 | 2.62 ± 0.29 | 20.82 ± 3.89 | Spearpole (10) |
|  | *Scarus rubroviolaceus* | 9 | 2.00 ± 0.00 | 31.14 ± 5.36 | Spearpole (9) |
| Detritivore | *Crenimugil crenilabis* | 10 | 2.29 ± 0.14 | 12.90 ± 6.62 | Thrownet (10) |
| Planktivore | *Caesio teres* | 10 | 3.40 ± 0.45 | 27.27 ± 1.84 | Spearpole (10) |
|  | *Caesio xanthonota* | 10 | 3.40 ± 0.45 | 26.12 ± 2.10 | Spearpole (10) |
|  | *Pterocaesio tile* | 1 | 3.33 ± 0.33 | 18 | Spearpole (1) |
| Corallivore | *Chaetodon trifasciatus* | 10 | 3.34 ± 0.61 | 9.67 ± 1.12 | Spearpole (10) |
| Invertivore | *Lethrinus enigmaticus* | 7 | 3.83 ± 0.58 | 36.31 ± 2.20 | Spearpole (1); Hook and Line (6) |
|  | *Lethrinus lentjan* | 10 | 3.94 ± 0.25 | 32.12 ± 7.01 | Spearpole (6); Hook and Line (4) |
|  | *Lethrinus nebulosus* | 10 | 3.76 ± 0.19 | 52.41 ± 8.81 | Hook and Line (10) |
|  | *Parupeneus macronemus* | 10 | 3.50 ± 0.37 | 15.62 ± 3.00 | Spearpole (10) |
| Reef Carnivore | *Cephalopholis sonnerati* | 2 | 3.81 ± 0.60 | 35.25 ± 5.87 | Hook and Line (2) |
|  | *Variola louti* | 10 | 4.33 ± 0.70 | 50.14 ± 4.90 | Spearpole (1); Hook and Line (9) |
| Reef Piscivore | *Aethaloperca rogaa* | 8 | 4.20 ± 0.71 | 31.95 ± 7.02 | Spearpole (8) |
|  | *Cephalopholis miniata* | 1 | 4.29 ± 0.51 | 25.5 | Spearpole (1) |
|  | *Lutjanus bohar* | 10 | 4.27 ± 0.50 | 36.84 ± 10.93 | Spearpole (4); Hook and Line (6) |
| Reef & Pelagic Carnivore | *Selar crumenophthalmus* | 10 | 3.81 ± 0.18 | 26.83 ± 1.45 | Hook and Line (10) |
| Pelagic Piscivore | *Katsuwonus pelamis* | 5 | 4.43 ± 0.47 | 48.80 ± 3.63 | Hook and Line (5) |
|  | *Sarda orientalis* | 1 | 4.21 ± 0.69 | 56 | Hook and Line (1) |
|  | *Thunnus albacares* | 8 | 4.41 ± 0.41 | 68.88 ± 10.43 | Hook and Line (7) |
|  | **Total** | **157** |  |  |  |

**Table S2** Summary of values of δ^15^N and δ^13^C, and ratios of C:N reported for lipid and urea extracted reef manta ray (*Mobula alfredi*) muscle tissues relative to sample collection year, sex and life stage class.

| **Year** | **Sex** | **Life Stage Class** | **n** | **δ^15^N (‰)** | **δ^15^N (min/max)** | **δ^13^C (‰)** | **δ^13^C min/max** | **C:N** |
| --- | --- | --- | --- | --- | --- | --- | --- | --- |
| 2016 | Female |  | **8** | 10.37 ± 0.48 | 9.67, 10.93 | -17.87 ± 1.02 | -18.98, -16.23 | 3.03 ± 0.16 |
|  |  | Juvenile | 3 | 10.44 ± 0.39 | 10.00, 10.75 | -18.28 ± 0.83 | -18.98, -17.36 | 3.07 ± 0.1 |
|  |  | Sub-adult | 4 | 10.31 ± 0.65 | 9.67, 10.93 | -17.98 ± 0.93 | -18.59, -16.61 | 3.06 ± 0.18 |
|  |  | Adult | 1 | 10.44 | - | -16.23 | - | 2.78 |
|  | Male |  | **5** | 10.64 ± 0.44 | 9.87, 10.95 | -17.94 ± 0.86 | -19.16, -16.89 | 3.03 ± 0.11 |
|  |  | Juvenile | 1 | 10.95 | - | -18.25 | - | 3.07 |
|  |  | Sub-adult | 1 | 9.87 | - | -18.00 | - | 3.03 |
|  |  | Adult | 3 | 10.79 ± 0.11 | 10.69, 10.91 | -17.82 ± 1.19 | -19.16, -16.89 | 3.02 ± 0.15 |
|  | Annual Average | - | 13 | 10.47 ± 0.47 | 9.67, 10.95 | -17.9 ± 0.92 | -19.16, -16.23 | 3.03 ± 0.14 |
| 2017 | Female |  | **16** | 10.84 ± 0.41 | 10.03, 11.42 | -18.8 ± 1.02 | -20.22, -16.53 | 3.17 ± 0.19 |
|  |  | Juvenile | 2 | 11.07 ± 0.23 | 10.91, 11.23 | -19.22 ± 0.06 | -19.27, -19.18 | 3.25 ± 0.03 |
|  |  | Sub-adult | 7 | 10.86 ± 0.55 | 10.03, 11.42 | -18.98 ± 1.02 | -20.22, -16.97 | 3.21 ± 0.21 |
|  |  | Adult | 7 | 10.76 ± 0.29 | 10.2, 11.03 | -18.5 ± 1.18 | -19.45, -16.53 | 3.10 ± 0.20 |
|  | Male |  | **20** | 11.04 ± 0.23 | 10.61, 11.48 | -19.05 ± 0.44 | -19.45, -17.77 | 3.18 ± 0.07 |
|  |  | Juvenile | 2 | 10.74 ± 0.19 | 10.61, 10.88 | -19.04 ± 0.09 | -19.11, -18.98 | 3.16 ± 0.06 |
|  |  | Sub-adult | 8 | 11.18 ± 0.22 | 10.89, 11.48 | -18.98 ± 0.53 | -19.41, -17.77 | 3.19 ± 0.08 |
|  |  | Adult | 10 | 11 ± 0.19 | 10.75, 11.31 | -19.11 ± 0.42 | -19.45, -18.09 | 3.18 ± 0.08 |
|  | Unknown | - | 1 | 10.66 | - | -17.33 ± 0 | - | 2.82 |
|  | Annual Average | - |  | 10.95 ± 0.34 | 10.03, 11.48 | -18.9 ± 0.79 | -20.22, -16.53 | 3.17 ± 0.15 |
| **Total Combined** | **-** | **-** | **50** | **10.82 ± 0.42** | **9.67, 11.48** | **-18.64 ± 0.93** | **-20.22, -16.23** | **3.13 ± 0.16** |

**Table S3** Summary of total (TA), core (SEA_c_) and overlapping trophic niche areas for male and female reef manta rays (*Mobula alfredi*) over two sampling years at D’Arros Island, Seychelles. Overlap is based upon ellipses encompassing 95% of the data.

| Sampling Year | Sex | n | TA | SEA_C_ | Total Trophic Overlap (%) |
| --- | --- | --- | --- | --- | --- |
| 2016 | Female | 8 | 2.09 | 1.44 | 71.59 |
|  | Male | 5 | 1.20 | 1.57 | 78.06 |
| 2017 | Female | 16 | 2.04 | 0.8 | 51.63 |
|  | Male | 20 | 0.80 | 0.34 | 89.34 |

**
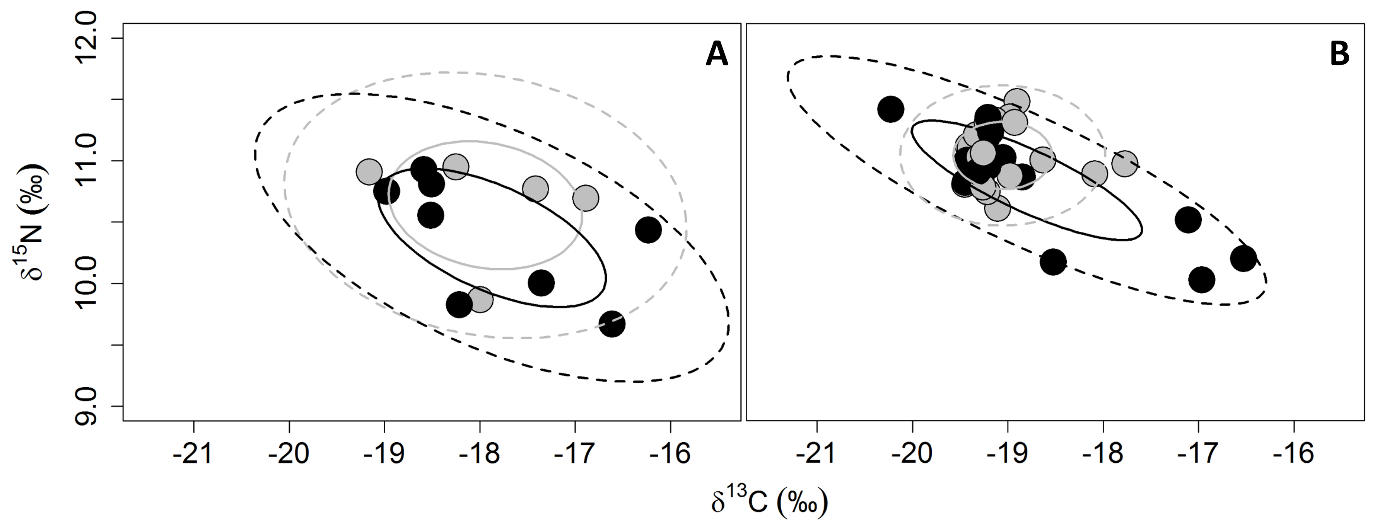
**

**Fig. S1** Isoscapes presenting the core (SEA_c_, solid lines) and total (TA, dashed lines) trophic niche areas of male (grey) and female (black) reef manta rays (*Mobula alfredi*) sampled at D’Arros Island, Seychelles, in November 2016 (A) and November 2017 (B).


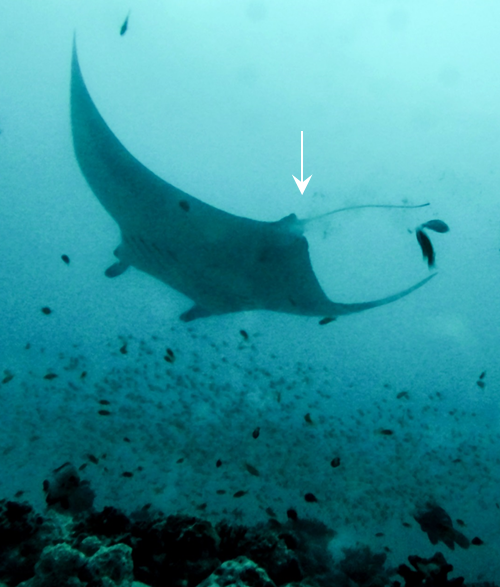


**Fig. S2** A reef manta ray (*Mobula alfredi*) defecates over the cleaning station at D’Arros Island, Seychelles. Arrow indicates faecal material.
